# Gold Plates Having Teeth Attached with Vulcanite

**Published:** 1905-06

**Authors:** F. J. M’Laren

**Affiliations:** New York, N. Y.


### GOLD PLATES HAVING TEETH ATTACHED WITH VULCANITE.[1]

BY F. J. M'LAREN, NEW YORK, N. Y.

ONE of the most useful dentures we have is the gold plate having the teeth attached with vulcanite. With all its advantages there is one great drawback,—namely, the necessity under ordinary circumstances of removing the rubber attachment if for any reason we are obliged to solder with gold, as when there is a fracture of the gold, or, in partial cases, if the addition of an isolated tooth or an extra clasp is required.

This not only involves a great amount of labor, but is always a source of dissatisfaction to the patient, because, in spite of every precaution, it is impossible to replace the teeth precisely as they were and to which the patient was accustomed.

In consequence I have found it necessary to devise certain methods of overcoming the difficulty, and believing that these are not ordinarily practised, I will endeavor to explain them. For instance, a case which frequently presents is one where an incisor has to be replaced, the plate being one having the bicuspids and

---

[1] Read before The New York Institute of Stomatology, February 7, 1905.

molars attached with vulcanite. The usual impression is taken with the plate in the mouth, and after pouring the model the selected tooth is backed and placed in position; a tail-piece is now fitted from the tooth reaching to and overlapping the plate. The tooth and tail-piece, being properly adjusted, are removed from the plate invested and soldered together. The tooth with the tail-piece soldered to it is now placed on the model and attached to the plate with hard wax. An investment is now made with which it is possible to have one portion of the plate cold, thereby protecting the rubber, and another part red hot to facilitate soldering. The investment is made in the following manner; first adapt pieces of iron to that portion of the plate under the rubber attachment; then use one or two large iron bars about the size of the ridge and extending about two inches back of the plate. If the bars do not come in sufficient contact with the plate, fill in the space between the gold and the bars with small nails, binding them all together with fine iron wire. Invest in plaster and asbestos, mainly asbestos. Make the investment in three sections separated from one another, one on either side protected by the metal, the third isolated from the plate but connected with the other two by means of small bent wires, leaving bare the portion to be soldered.

Place the case on a retort frame, exposing the isolated tooth to the heat of a Bunsen burner, being careful not to permit the heat to touch the part protecting the rubber. While the tooth is being heated use a bulb syringe to drop a little cold water on the side investments. This will allow the front tooth to become red for soldering, while the main plate will be perfectly cold. When the tooth becomes red transfer the investment to a soldering pan and have an assistant apply water constantly to the side investments and iron bars while the heat from the blowpipe is concentrated on the point to be soldered. Apply the heat rapidly, and do not for a moment allow the plate to become cold until soldering is completed.

When a plate has been split, reinforce with a thin piece of gold held in place by means of small clamps made of thin iron wire or other material, and place 18-carat filings between the piece of gold and the main plate.

For applying a clasp close to the vulcanite, it may be necessary to cut away part of the rubber in order to get a sufficient thickness of non-conducting material, usually asbestos cloth, and where the clasp is very close to the rubber there may be danger of scorching, but the trouble of replacing a small surface of vulcanite is slight as compared with taking off the teeth and disturbing the articulation. Too much stress cannot be placed upon the advisability of using a concentrated flame, and while, as a rule, I use the ordinary foot-power blow-pipe, in many instances it will be found necessary to attach a cylinder of nitrous oxide gas to the air-tube of the blow-pipe. This gives an intense concentrated heat to the point of soldering.

I have successfully used the foregoing methods for several years, and hope that I have been able to make myself clear, as these suggestions may help to eliminate one of the many annoyances in the existence of a busy dentist.

